# Anti-Inflammatory and Anti-Angiogenesis Effects of Verapamil on Rat Air Pouch Inflammation Model

**DOI:** 10.15171/apb.2017.070

**Published:** 2017-12-31

**Authors:** Tahereh Eteraf-Oskouei, Sevda Mikaily Mirak, Moslem Najafi

**Affiliations:** ^1^Department of Pharmacology and Toxicology, Faculty of Pharmacy, Tabriz University of Medical Sciences, Tabriz, Iran.; ^2^Student Research Committee, Faculty of Pharmacy, Tabriz University of Medical Sciences, Tabriz, Iran.

**Keywords:** Verapamil, Air-Pouch, Inflammation, Angiogenesis, VEGF, IL-1β

## Abstract

***Purpose:*** In the present study, the effects of verapamil on inflammation and angiogenesis in air pouch model were studied.

***Methods:*** To create a model of inflammation in the rats, on days 1 and 3 sterile air, and on the sixth day, carrageenan was injected into the pouch subcutaneously. Normal saline as control, diclofenac sodium and dexamethasone as standards and verapamil (0.05, 0.1 and 0.2mg/rat) was injected into the pouch simultaneously with carrageenan and as well as 24 and 48 hours later. After 72 hours, volume of exudate, the leukocytes count, concentration of VEGF and IL-1ß, granulomatous tissue weight, histopathological changes and angiogenesis were considered.

***Results:*** Verapamil significantly reduced leukocyte accumulation in all doses, but effect of 0.1mg/rat was more significant (P<0.001). The exudate volume and granulomatous tissue weight was reduced with all doses, especially 0.1mg/rat (P<0.01). Doses 0.05, 0.1 and 0.2mg/rat of verapamil compared with the control group (carrageenan) led to a reduction in the amount of hemoglobin in the tissue as the angiogenesis indicator (P<0.001, P<0.01 and P<0.05, respectively). VEGF level of exudate was reduced by doses of 0.05 and 0.1mg/rat (P<0.05). In addition, IL-1β concentration was lowered by 0.1mg/rat of verapamil (P<0.05). Histopathological changes, severity of granulomatous inflammation, granulomatous tissue cell density and angiogenesis in verapamil group were markedly lower compared to carrageenan group.

***Conclusion:*** Verapamil has significant anti-inflammatory and anti-angiogenesis effects in the air pouch model probably due to attenuation effects of verapamil on IL-1β and VEGF.

## Introduction

Inflammation is immune response to infection or tissue injury and plays a major role in the pathogenesis of diseases such as arthritis, cancer, cardiovascular and neurodegenerative diseases.^1^ Inflammation leads to activation of local tissues and release of intermediates of them that ultimately causes vasodilatation and increased vascular permeability, swelling and pain fibers activity.^2^ Induction of acute inflammation is the main method employed by the innate immunity system to fight against infections and tissue damages that can be created within minutes to hours and continues to days. Proinflammatory TNF-α, IL-1 and IL-6 cytokines play an important role in activating the inflammatory cells.^3^ If the infection is not removed or the tissue damage is prolonged, chronic inflammation continues after acute inflammation.^4^ Chronic inflammation sites often undergo tissue regeneration along with angiogenesis and fibrosis.^5^

Glucocorticosteroids and non-steroidal anti-inflammatory drugs (NSAIDs) are the most common anti-inflammatory drugs,^6^ but cause serious side effects in long-term use. So today, attempts are made to provide drugs, in addition to suitable anti-inflammatory effects of which, cause the least side effects.^7^

Calcium ions play an important role in the synthesis and release of chemical mediators of inflammation.^8^ Calcium leads to the activation of nitric oxide synthase, phospholipase A_2_ (PLA_2_) and phospholipase C (PLC) enzymes. PLA_2_ causes a release of arachidonic acid, which is a precursor of prostaglandin synthesis, leukotrienes and thromboxanes.^9^ Inflammatory leukocytes perform a wide range of functional responses such as degranulation, production of superoxide, nitric oxide (NO) and TNF-α through calcium ion. Thus, inhibition of calcium influx may be an important factor in reducing leukocyte activation.^10^

Calcium channel blockers (CCBs) such as verapamil exert their cardiovascular effects by blocking voltage-dependent L-type calcium channels.^11^ Increased calcium influx through type L calcium channels causes the activation of various signaling cascades, such as reactive oxygen and nitrogen species and activation of proinflammatory cytokines.^12^ According to previous studies, verapamil can inhibit the production of inflammatory cytokines such as TNF-α, IL-1 and IL-6 and increase plasma levels of inflammatory cytokines such as IL-10.^13,14^

There is no report on exact effects of verapamil on the inflammatory and angiogenesis parameters in the air pouch model, so the possible anti-inflammatory and anti-angiogenesis effects of this drug were studied in rats in this study.

## Materials and Methods

### Animals

In this study, male Wistar rats (200-250g) were used. The animals had free access to food and water under standard temperature conditions of 21±3 °C and were kept in 12 hours light and 12 hours of darkness.

### Air pouch model of inflammation 

The hair of animals is shaved over the whole dorsal region after being anesthetized and 20ml of sterile air was injected subcutaneously. Three days later, 10ml of air was injected into the above region. One ml of 1% carrageenan solution was injected into the pouch on the sixth day after the initial air injection.

### Studied groups

The rats were randomly divided into eight groups:

**1&2- Normal saline control group and control carrageenan**: These groups respectively received 1ml normal saline or carrageenan (intra-pouch) after creation of pouch on the sixth day. Then, 1ml normal saline was injected into the pouch immediately before and 24 and 48 hours later.

**3. Diclofenac sodium positive control group**: To compare the effect of verapamil with NSAIDs (as standard) drugs, this group received 1mg diclofenac at volume of 1ml on the sixth day after formation of pouch, immediately before the injection of carrageenan and 24 and 48 hours later.

**4. Dexamethasone positive control group:** To compare the effects of verapamil with corticosteroids (as standard), this group, similar to group 3 received 0.4mg dexamethasone (1ml, intra-pouch).

**5-8. Verapamil treatment groups**: These groups after pouch formation on the sixth day, immediately before the injection of carrageenan and 24 and 48 hours later, 0.05, 0.1, 0.2 and 2.5mg/rat of verapamil (1ml) was injected intra-pouch.

### Studied inflammatory parameters

#### Determining exudate volume and counting the number of leukocytes

Three days after the injection of carrageenan, the rats were sacrificed and the inflammatory exudate was removed and its volume was measured. Some of the exudate was later poured into the test tube containing EDTA and leukocyte count was carried on by Neubauer slide after mixing and dilution under light microscope (Olympus, Japan).

#### Measurement of VEGF and IL-1ß in exudate

Three days after the induction of inflammation, exudate collected was centrifuged at 1000g and 4°C for 10 minutes. The cell-free supernatant was used to determine the amount of VEGF and IL-1ß using ELISA according to the manufacturer's instructions.

#### Angiogenesis evaluation

Angiogenesis was determined using the method described previously (Ghosh et al.) with a little change. Granulomatous tissue was initially removed, washed with PBS solution (pH=7.4) and dried. It was later cut into small pieces, then Drabkin solution (ZistChem Diagnostics, Iran) was added and homogenized at 15000 for 5 minutes at ice bed by a homogenizer (HO4 AP-Edmund Bϋhler, B. Braun, Germany). The homogenized tissue was centrifuged at 10000 RCF and 4°C for 30 minutes. Subsequently, 1ml of supernatant was later mixed with 4ml of Drabkin solution and after being smooth out by millipore filters (0.22µ), the amount of hemoglobin was determined as a marker of angiogenesis using hemoglobin standard curve by UV spectrophotometer at 540nm in accordance with the kit manufacturer instructions.^15^

#### Granulomatous tissue weighing and evaluating its histopathological changes

Three days after the injection of carrageenan, the animals were sacrificed and the outer skin of the formed cavity was cut and the pouch was isolated from the surrounding tissues and weighed. Granulomatous tissue formed around pouch was fixed in 10% formalin and was later cut by a microtome (Lieits, Germany) with diameter of 5.6 microns after paraffin dehydration and molding process and was finally placed on a slide. After deparaffinization, the slides were stained by hematoxylin-eosin and the histopathological changes, including granulomatous intensity, polymorphonuclear density, macrophage density and angiogenesis intensity were evaluated using the light microscope.

#### Statistical analysis of data

The results were reported as mean±SEM and SPSS v. 17 was used for statistical analysis. Independent-samples t-test was used to compare between normal saline and carrageenan control groups to ensure the validity of the formed inflammation. The other groups were compared by one-way ANOVA and post hoc LSD test. P-value of <0.05 was considered statistically significant.

## Results

First, to ensure the validity of the inflammation formed in the air pouch, a comparison was made on the results obtained in the normal saline control and carrageenan groups. All studied parameters in the carrageenan control group were significantly increased compared with normal saline group (P<0.001).

### Effect of verapamil on leukocyte accumulation

**Figure 1:**
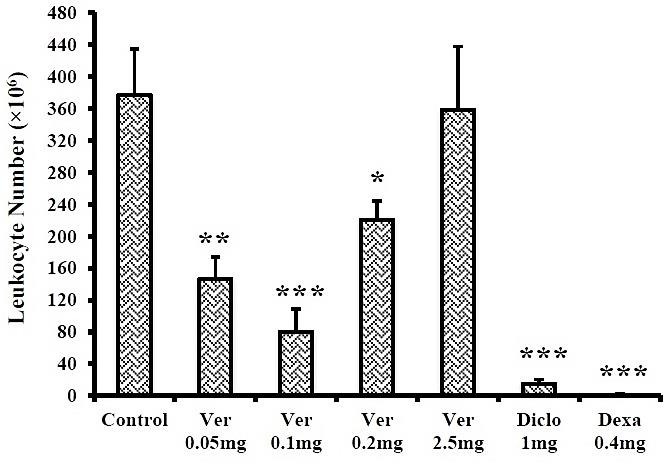
Effects of verapamil on the leukocyte accumulation in the pouch fluid in male rats (n≥6). Data are presented as mean±SEM. * P<0.05, ** P<0.01, *** P<0.001 compared to the control carrageenan. Ver: Verapamil, Diclo: Diclofenac sodium, Dexa: Dexamethasone.

As can be seen in Figure 1, the total number of leukocytes in the diclofenac and dexamethasone groups was 15.35±5.18 and 1.66±0.49 million, respectively. Verapamil by doses 0.05, 0.1 and 0.2mg/rat reduced leukocytes compared with the carrageenan group (P<0.01, P<0.001 and P<0.05, respectively), but 2.5mg/rat of verapamil did not create a significant decrease. In this parameter, all verapamil doses showed significant difference with diclofenac and dexamethasone.

### Effect of verapamil on the volume of exudate and granulomatous tissue weight

**Figure 2:**
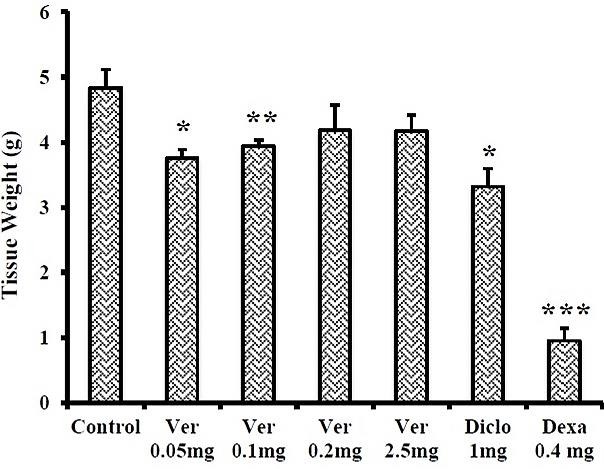
Effects of verapamil on the granulomatous tissue weight in male rats (n≥6). Data are presented as mean±SEM. * P<0.05, ** P<0.01, *** P<0.001 compared to the control carrageenan. Ver: Verapamil, Diclo: Diclofenac sodium, Dexa: Dexamethasone.

Exudate volume in diclofenac and dexamethasone groups was 4.61±0.56 and 2.27±0.23ml, respectively. The same volume was obtained 6.65±0.66, 4.74±0.92, 6.38±0.43 and 6.54±0.37ml at verapamil doses of 0.05, 0.1, 0.2 and 2.5mg/rat, respectively. Only 0.1mg/rat of verapamil led to a significant reduction as compared to carrageenan (P<0.01). In addition, diclofenac, dexamethasone, 0.1 and 0.05mg/rat verapamil doses significantly reduced the granulomatous tissue weight compared with carrageenan (Figure 2).

### Effect of verapamil on granulation tissue angiogenesis

In order to evaluate angiogenesis, tissue hemoglobin was measured as a marker of angiogenesis. Verapamil by doses of 0.05, 0.1 and 0.2 mg/rat significantly reduced angiogenesis from the carrageenan control group value (291.0±10.5) to 154.5±12.0 (P<0.001), 184.7±28.0 (P<0.01) and 241.2±18.5 mg/100g tissue (P<0.05), respectively. In addition, compared to the control group, angiogenesis was decreased by diclofenac and dexamethasone to 170.4±17.4 (P<0.001) and 32.3±7.9 (P<0.001), respectively.

### Effect of verapamil on the amount of exudate VEGF 

Considering the importance of VEGF protein in angiogenesis, as well as to confirm the data obtained from angiogenesis, its amount was measured in inflammatory exudates using more effective verapamil doses. VEGF levels in the carrageenan and diclofenac groups, 0.05, 0.1mg/rat verapamil groups were 72.804±6.736, 25.563±6.027, 46.254±3.543 and 48.441±6.098ng, respectively. Diclofenac at P<0.001 and both verapamil doses at P<0.05 reduced VEGF levels compared with carrageenan group.

### Effect of verapamil on the amount of exudate IL-1β 

The amount of IL-1β was 7.432±0.487pg in the carrageenan group, which was reduced to 3.547±0.052pg when diclofenac was used. Also, 0.1 dose of verapamil (the most effective dose to reduce all inflammatory parameters) decreased IL-1β level to 4.683±0.622pg that was significant compared with the carrageenan group (P<0.05).

### Comparative study of verapamil effects on granulation tissue angiogenesis and VEGF level 

**Figure 3:**
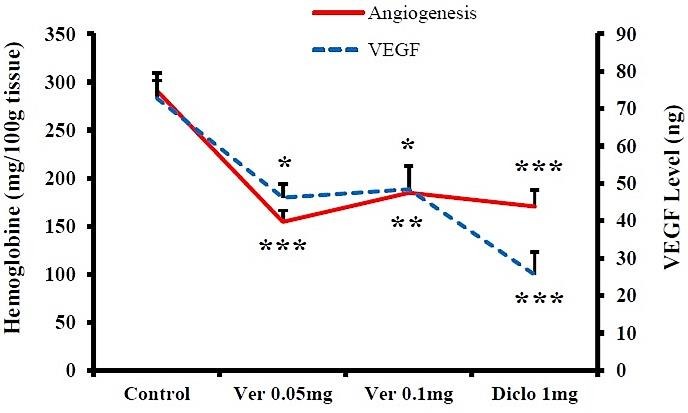
Comparative study of verapamil effects on granulation tissue angiogenesis and VEGF in male rats (n≥6). Data are presented as mean±SEM. * P<0.05, ** P<0.01, *** P<0.001 compared to the control carrageenan. Ver: Verapamil, Diclo: Diclofenac sodium.

VEGF is one of the most important factors in stimulating angiogenesis and exerts its stimulatory effects by increasing migration and proliferation of endothelial cells and formation of vascular network.^16^ Given the key role of VEGF in the angiogenesis process, the effects of verapamil on angiogenesis and VEGF were compared in Figure 3. Verapamil could reduce angiogenesis and VEGF level using a similar pattern.

### Effect of verapamil on histopathological changes of granulomatous tissue

**Figure 4:**
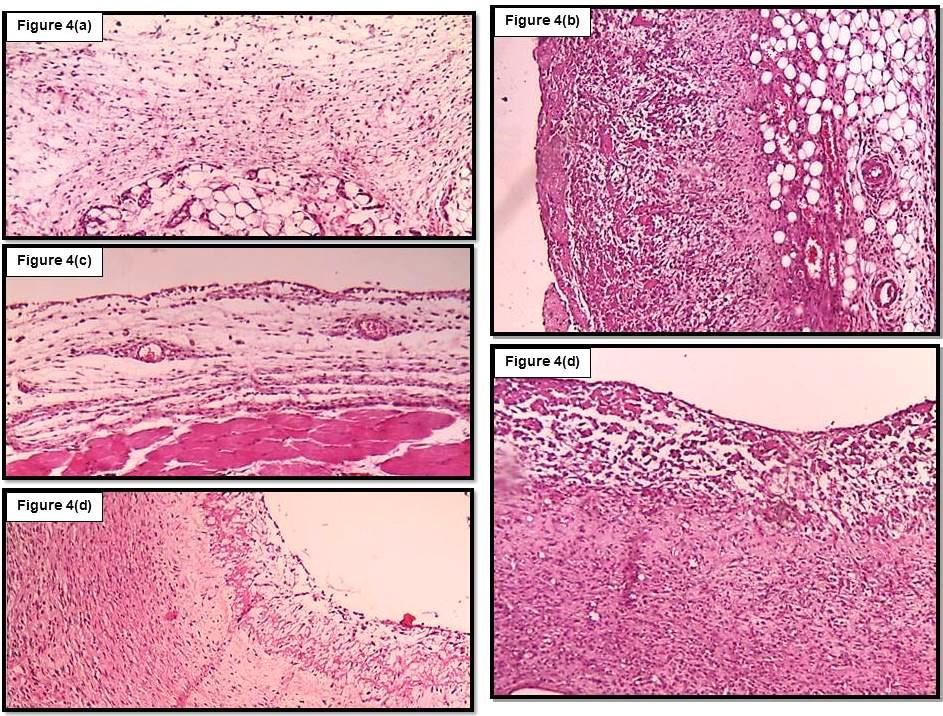
Histopathological changes in granulomatous tissue in different groups. 4(a): normal saline group; minimal inflammation and small cell accumulation and almost no trace of granulomatous tissue and angiogenesis. 4(b): carrageenan group; a large number of macrophages and many congested young vessels with very intense granulomatous inflammation. 4(c): dexamethasone group; the severity of granulomatosis and inflammation are clearly reduced 4(d): diclofenac group; moderate granulomatous inflammation with low macrophages and angiogenesis compared to carrageenan group. 4(e): verapamil 0.05mg; inflammation and granulomatous inflammation are reduced compared to the carrageenan group. A significant reduction in cell density and angiogenesis is also observed.

Granulomatous intensity, polymorphonuclear density, macrophage density and angiogenesis intensity in normal saline and carrageenan control groups was evaluated mild and very severe, respectively. In the groups treated with verapamil, anti-inflammatory and anti-angiogenesis effects were shown to be associated with significant decrease in these parameters that is comparable to the group receiving diclofenac. The most decreasing effect on these parameters was observed in the dexamethasone group. Figure 4 shows an example of histological changes in granulomatous tissue in different groups.

## Discussion

Calcium ions play a major role in the synthesis and release of inflammatory mediators^11^ and CCBs can be used to study its inflammatory role.^9^ The present study aimed to examine the effect of verapamil, as L-type calcium channel blocker, on inflammatory parameters and angiogenesis in air pouch experimental model of inflammation.

The leukocyte accumulation was significantly decreased using verapamil, especially at a dose of 0.1mg/rat. Bilici et al. in their study on the effects of mibefradil (a new CCB) on histamine-induced inflammation in the rat paw showed that the agent is able to reduce leukocyte accumulation.^17^ Diltiazem is another CCB drugs that prevented inflammation induced by yeast injection into the pouch and the leukocyte accumulation.^8^ The observed effects of verapamil in reducing the accumulation of leukocytes in this study was consistent with the results of two above studies.

Proinflammatory cytokines, including TNF-α and IL-1 play an important role in the activation of endothelial cells by increasing the expression of adhesion molecules of ICAM-1 and VCAM-1. Mutual reactions of adhesion molecules and leukocyte ligands are very important in leukocyte recruitment.^18^ In BALB/c mice that underwent an intraperitoneal injection of LPS, an inflammation-causing substance, verapamil reduced serum levels of TNF-α.^19^ A study on TNF-α-stimulated human endothelial cells showed that verapamil reduced binding rate of leukocytes to endothelial cells. Also, verapamil reduced the expression of VCAM-1 in IL-1β-stimulated endothelial cells.^20^ Our results showed that the concentration of IL-1β was significantly reduced when verapamil 0.1mg/rat was applied. It seems that the inhibitory effects of verapamil on serum levels of TNF-α, the expression of VCAM-1 and IL-1β in the exudates can play a role in decreasing leukocyte recruitment.

Calcium is an important biochemical mediator in the activation of macrophages, calcium channel blockers such as verapamil and nifedipine can prevent the activation of macrophages.^21^ Plasminogen also plays an important role in the recruitment of macrophages to the inflammation site.^22,23^ Increased intracellular calcium results in overexpression of plasminogen receptor on the surface of macrophages differentiated monocytes and verapamil or nifedipine inhibits this process.^23^ PLA_2_ enzyme needs calcium in order to exert its effect on arachidonic acid.^24^ Verapamil by blocking calcium channels can prevent the release of arachidonic acid and prostaglandin synthesis and leukotriene via inhibiting PLA_2_ activity.^24^

Following injection of carrageenan, vascular permeability is increased by the release of mediators such as histamine, serotonin, prostaglandins, leukotrienes and NO, as a result of which inflammatory exudates are formed.^25-27^ Prostaglandin E_2_ and leukotrienes C_4_, D_4_ and E_4_ are involved in this process respectively through vasodilatation and increased vascular permeability.^28^ In this study, verapamil reduced the volume of exudate. In another study, verapamil has been effective on inflammation caused by injecting carrageenan in the rat paw and reduced the resulting edema.^9^ Studies on dihydropyridine calcium antagonists such as amlodipine and nicardipine showed that these drugs significantly reduced the edema in carrageenan-induced inflammation in the rat paw.^27^ Probably, inhibition of eicosanoid synthesis by verapamil prevents the formation of inflammatory exudates.

Granulation tissue formation is one of the features of chronic inflammation and accumulation of fibroblasts and macrophages, collagen synthesis and angiogenesis are important characteristics of granulation tissue.^29^ Calmodulin-calcium complex leads to the activation of various enzymes that are involved in the proliferation and activation of fibroblasts.^30^ Maybe, verapamil prevents granulation tissue development by inhibiting the activity of fibroblasts.

In the present study, granuloma tissue weight was decreased with 0.05 and 0.1mg of verapamil. However, in Dengiz et al.'s study, verapamil (10mg/kg) did not reduce the granulomatous tissue weight in granulomatosis inflammation induced by subcutaneous implantation of cotton pellet in rat shoulder,^31^ but the same dose of nicardipine caused significant weight loss in granulomatous tissue.^27^ In *in vitro*, cytokines derived from macrophages (IL-1 and TNF-α) in the formation of granulation tissue are very important. The inhibitory effect of verapamil on TNF-α^11,19,32^ and IL-1 can prevent development of granulomatous tissue. Our study also showed the inhibitory effect of verapamil on the amount of IL-1β in inflammatory exudates.

Several studies have shown that NO plays an important role in inflammation induced by carrageenan.^27^ In the carrageenan-induced inflammation, NO is derived from vascular endothelial cells^33^ and its synthesis pathway is calcium dependent, so inhibiting the NO synthesis by verapamil is another potential mechanism to reduce the inflammatory exudates. Also, NO produced by the macrophages is effective on the formation of granulomatous tissue.^34^ Studies on LPS-stimulated microglia cells showed that NO production was significantly reduced by verapamil.^11^

A distinctive feature of granulomatous tissue is angiogenesis,^29^ on which the persistence of chronic inflammation depends and angiogenesis inhibition can prevent inflammation.^35^ In the present study, verapamil could prevent angiogenesis of granulomatous tissue. VEGF is one of the most important factors stimulating angiogenesis and its inhibition may be a therapeutic target to decrease angiogenesis.^16^ The results of this study showed that verapamil, like angiogenesis, caused significant reduction in VEGF levels of exudate. In fact, angiogenesis and VEGF reduction pattern is very similar and indicates a significant correlation between angiogenesis and VEGF. VEGF reduction by verapamil may be an important mechanism to inhibit angiogenesis. In a study, Giuglian et al. demonstrated that verapamil prevented VEGF production by fibroblasts in hypoxic conditions.^36^ Inhibition of TNF-α,^11,19,32^ decreased NO production by macrophages,^11^ inhibition of prostaglandin E_2_ synthesis,^24^ and decreased matrix metalloproteinase activity in blood mononuclear cells^37^ are all the possible mechanisms of verapamil that are involved in decreasing angiogenesis in addition to reducing VEGF levels.

Verapamil reduction pattern was just bell-shape in the case of inflammatory parameters and angiogenesis so that the maximum effect was observed at the middle dose and the anti-inflammatory effect was reduced in higher and lower doses. Such bell-shaped dose-response curve was previously reported for verapamil in cardiovascular system,^38^ including in the treatment of ischemic arrhythmias.^39,40^ The mechanism of such dose-response curve is unknown, but probably reflects the complex pharmacology of verapamil.^41^

## Conclusion

The findings of this study showed that verapamil had significant anti-inflammatory and anti-angiogenesis effects in the air pouch model of inflammation, which is likely due to attenuation effects of verapamil on IL-1β and VEGF.

## Acknowledgments

This study was supported by Student Research Committee, Tabriz University of Medical Sciences, Tabriz, Iran. The authors would like to thank Drug Applied Research Center, Tabriz University of Medical Sciences for providing laboratory facilities and supporting this work. Verapamil was gifted by Alborz Daru Pharmaceutical Company, Iran.

## Ethical Issues

This study was carried out in accordance with the principles and rules maintenance and use of laboratory animals approved by the Ethics Committee of Tabriz University of Medical Sciences.

## Conflict of Interest

No potential conflicts of interest were disclosed.
